# Genome Survey and SSR Analysis of *Camellia nitidissima* Chi (Theaceae)

**DOI:** 10.1155/2022/5417970

**Published:** 2022-11-02

**Authors:** Yu Bai, Lin Ye, Kang Yang, Hui Wang

**Affiliations:** ^1^College of Mathematics and Information Science, Guiyang University, Guiyang 550005, China; ^2^School of Electronic & Communication Engineering, Guiyang University, Guiyang 550005, China; ^3^Guizhou Provincial Key Laboratory for Rare Animal and Economic Insects of the Mountainous Region, Guiyang University, Guiyang 550005, China

## Abstract

*Camellia nitidissima* Chi (CNC), a species of golden *Camellia*, is well known as “the queen of camellias.” It is an ornamental, medicinal, and edible plant grown in China. In this study, we conducted a genome survey sequencing analysis and simple sequence repeat (SSR) identification of CNC using the Illumina sequencing platform. The 21-mer analysis predicted its genome size to be 2,778.82 Mb, with heterozygosity and repetition rates of 1.42% and 65.27%, respectively. The CNC genome sequences were assembled into 9,399,197 scaffolds, covering ∼2,910 Mb and an N50 of 869 base pair. Its genomic characteristics were found to be similar to those of *Camellia oleifera*. In addition, 1,940,616 SSRs were identified from the genome data, including mono-(61.85%), di-(28.71%), tri-(6.51%), tetra-(1.85%), penta-(0.57%), and hexanucleotide motifs (0.51%). We believe these data will provide a useful foundation for the development of novel molecular markers for CNC as well as for further whole-genome sequencing of CNC.

## 1. Introduction

*Camellia nitidissima* Chi (CNC), a species of golden *Camellia*, is well known as “the queen of camellias” [1, 2]. It is largely grown in Guangxi province, China and has been introduced into Fujian province, China. *C*. *nitidissima* is a well-known ornamental plant because of its golden yellow flowers [2] that contain several flavonoids and polyphenols [3]. In addition, *C*. *nitidissima* is a well-known medicinal and edible plant in China [4]. The leaves and flowers of CNC have antioxidant and antimicrobial activities [1, 5–7] and are used as pancreatic lipase inhibitors [8] and potential anticancer drugs for gastric and colon cancers [9, 10].

Simple sequence repeats (SSRs), also known as microsatellites, are stretches of DNA consisting of tandemly repeated short units, 1‒6 base pairs (bp) in length [11], which have been identified and characterized in the genus *Camellia*. In the last 15 years, several SSRs markers have been developed from microRNA (miRNA), mRNA, genome, and chloroplast sequences to study the genetic variation and population structure in different genera of *Camellia* [12–41], such as *C. sinensis*, *C. osmanthus*, *C. vietnamensis*, *C. gauchowensis*, *C. huana*, *C. sasanqua*, *C. oleifera*, *C. japonica,* and *C. reticulata*. In the last three years, SSR markers in the genus *Camellia* have emerged as a highly interesting research topic, with at least 14 studies on SSR markers [28–41], including both genome-wide SSR markers and SSR identification of single resistance genes, gene families, whole transcription factors, and the development of SSR databases. For example, an SSR marker was used as a molecular marker to tag the blister blight disease-resistance trait of *C. sinensis* [29, 35]. Similarly, 72 SSR loci were detected in 14 and 15 phospholipase D gene families of *C. sinensis* for marker-assisted selection of resistance genes [37]. In addition, 3,687 SSR loci from 2,776 transcripts of transcription factor gene transcripts were identified for potential implications in trait dissection [40]. TeaMiD was developed for simple sequence repeat markers of *C. sinensis,* including 935,547 SSRs [41].

However, only 15 polymorphic microsatellite loci have been isolated and characterized from *C. nitidissima* [42]. Genome-wide SSR markers of *C. nitidissima* have not been identified because of a lack of genome sequences. Therefore, it is necessary to estimate the genome size and identify genome-wide SSRs in *C. nitidissima* using next-generation sequencing (NGS), which will be useful for further whole-genome sequencing and assessing genetic diversity within and among populations.

## 2. Materials and Methods

### 2.1. Plant Materials

CNC was obtained from Longyan City, Fujian Province, China. The leaf tissue was immediately collected from CNC, washed in sterile phosphate-buffered saline (PBS), frozen in liquid nitrogen, and stored at −80°C for further analysis.

### 2.2. DNA Extraction and Genome Sequencing

The total DNA of CNC was isolated using the cetyltrimethylammonium bromide (CTAB) DNA extraction protocol [43, 44]. The purity and concentration of the obtained gDNA were tested using a NanoPhotometer® spectrophotometer (Implen, CA, USA) and a Qubit® 2.0 fluorometer (Life Technologies, CA, USA), respectively [45]. Sequencing libraries for the quality-checked gDNA were generated using a TrueLib DNA Library Rapid Prep Kit for Illumina sequencing (Illumina, Inc., CA, USA) [45]. The libraries were subjected to size distribution analysis using an Agilent 2100 bioanalyzer (Agilent Technologies, Inc., CA, USA), followed by a real-time PCR quantitative test [45]. The successfully generated libraries were sequenced using an Illumina NovaSeq 6000 platform (Illumina, Inc., CA, USA), and 150-bp paired-end reads with an insert of approximately 350 bp that was generated [45].

### 2.3. DNA Data Cleaning and Genome Assessment

The obtained raw reads were filtered to obtain clean reads using trimmomatic version 0.36 (https://www.usadellab.org/cms/index.php?page=trimmomatic) [46]. The quality control (QC) standards of reads from DNA were as follows:

1. Trimming adapter sequences,
2. Trimming low quality or 3 bases (below quality 3) in the front of the reads,
3. Trimming low quality or 3 bases (below quality 3) in the tail region for reads,
4. Scan the read with a 4-base wide sliding window, cutting when the average quality per base drops below 15,
5. Removing reads with <51 bases.

To estimate the status of contamination from other species, 20,000 reads (10,000 reads from read 1 and 10,000 reads from read 2) were randomly selected from the resulting high-quality cleaned reads against the NCBI nonredundant nucleotide sequence (NT) database using the blastn software version 2.2.28 (https://blast.ncbi.nlm.nih.gov/Blast.cgi) [47, 48], with an E-value threshold of 1 × 10^−5^.

The resulting high-quality clean reads from DNA sequencing were subjected to K-mers analysis using Jellyfish version 2.3.0 (https://genome.umd.edu/jellyfish.html) [49] with savings in the hash-only canonical K-mers (−C) and K-mers values (−m 19, 21, and 23). Genome size, heterozygosity ratio, read duplication ratio, and read error ratio were estimated using GenomeScope version 2.0 (https://qb.cshl.edu/genomescope/) [50] with R version 4.1.3. The repeat rate was estimated as the percentage of the number of K-mers after a 1.8 fold in the main peak depth over the total number of K-mers.

### 2.4. Genome Assembly, GC Content Analysis, SSRs Identification, And Primer Design

The CNC genome was assembled using SOAPdenovo2 version 2.40 (https://github.com/aquaskyline/SOAPdenovo2) [51] with a K-mers value of 51 and other default settings. The GC content was calculated using contigs longer than 500 bp. SSRs were identified using MISA version 2.1 [11] with default parameters (SSR pattern: 1‒10, 2‒6, 3‒5, 4‒5, 5‒5, and 6‒5; the maximum length of sequence between two SSRs to register as a compound SSR was 100 bp). Primer pairs were designed using Primer3 version 2.6.1 [52], which were selected to meet the following criteria: the expected PCR product size ranged from 100 to 280 bp; primer length ranged from 18 to 23 bp (optimum length: 20 bp); primer melting temperature ranged from 57.0 to 60°C (optimum temperature: 5°C); and primer GC content ranged from 40 to 70%.

## 3. Results

### 3.1. Sequencing and QC of CNC

Approximately 343.06 Gb of high-quality, clean reads were obtained using the trimmomatic software [46] from approximately 382.21 Gb of raw reads using the Illumina NovaSep platform for the CNC genome survey (Table 1). The Q20, Q30, and GC content values of the clean reads were 95.67%, 89.52%, and 37%, respectively. The top six species from 20,000 randomly selected clean reads in the NT database were *C*. *sinensis* (2.26%), *C*. *taliensis* (0.17%), *Vitis vinifera* (0.11%), *Helianthus maximiliani* (0.05%), *C*. *yunnanensis* (0.05%), and *C*. *pitardii* (0.03%), indicating that there was no contamination from other species.

### 3.2. Genome Assessment

**Figure 1:**
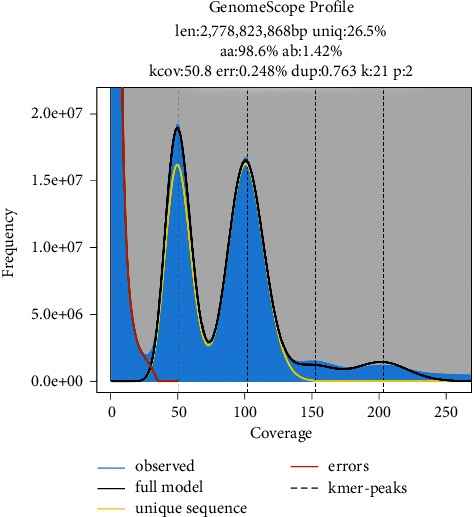
21-mers distribution of the CNC genome. Blue bars represent the observed K-mer distribution; the black line represents the modeled distribution without the error K-mers (red line), up to the maximum K-mer coverage specified in the model (yellow line). Len, estimated total genome length; Uniq, unique portion of the genome (not repetitive); het, heterozygosity rate; Kcov, mean K-mer coverage for heterozygous bases; Err, error rate; and Dup, duplication rate.

We estimated the CNC genome size using the K-mers value (*K* = 19, 21, and 23) (Table 2). According to the 21-mers recommendation [50], the CNC genome size and K-mer depth were 2, 778, 823, 868 bp and 101, respectively (Figure 1). The error and duplication rates of the reads were 0.248% and 0.706%, respectively. The heterozygosity and repeat rates of the sequences were 1.42% and 65.27%, respectively. The heterozygous peak K-mer frequency was 50, which indicates that the CNC genome has high heterozygosity (heterozygosity rate ≥0.8%) and high repetition (repetition rate ≥50%).

### 3.3. Genome Assembly and GC Content Analysis

**Figure 2:**
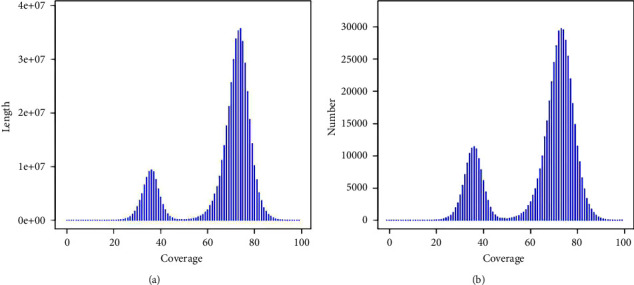
Contig distribution of the CNC genome. (a) Distribution graph of contig coverage depth and length and (b) distribution graph of the CNC contig coverage depth and number. In the figure, the peak with the highest distribution was the main peak. The heterozygosity of the genome was judged according to the peak of 1/2 position before the main peak.

**Figure 3:**
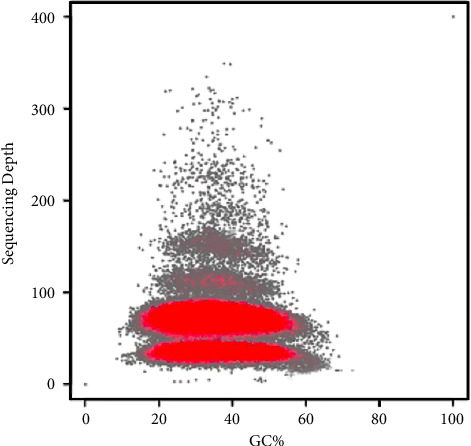
GC content and depth correlation graph of the CNC genome. The red part represents the dense part of the points in the scatter plot.

The clean reads were assembled into 9,994,482 contigs and 9,399,197 scaffolds using the SOAPdenovo software with 51-mers value (Table 3). The total length of the contigs and scaffolds was 2,844,296,380 and 2,910,885,755 bp, respectively. According to the significant peaks of the CNC contig distribution (Figure 2), the peak located halfway in front of the main peak was the heterozygous peak [44], which also proved the existence of high heterozygosity in the CNC genome. Because of the high heterozygosity, the assembled haploid genome was larger than predicted. The maximum lengths of the contigs and scaffolds were 73,907 bp and 88,303 bp, respectively. The N50 lengths of the contigs and scaffolds were 649 bp and 869 bp, respectively. The GC contents of the contigs and scaffolds were 36.00% and 34.00%, respectively. The GC content of the scaffolds was lower than that of the contigs owing to the presence of an N base. The GC depth analysis (Figure 3) indicated that the GC content of the windows was mostly concentrated in the range of 20‒60%, which did not show any apparent abnormalities or GC bias [44]. The GC depth distribution was divided into two layers, which indicated the high heterozygosity of the CNC genome.

### 3.4. SSR Identification

A total of 1,940,616 SSRs were identified from 1,026,855 scaffolds in the CNC genome, including 346,619 SSRs involved in compound formation. In total, 332,308 scaffolds contained more than one SSR. The largest group of motifs was mononucleotide repeats (1,200,317 motifs; 61.85%). This was followed by dinucleotide (557,218 motifs, 28.71%), trinucleotide (126,286 motifs, 6.51%), tetranucleotide (35,890 motifs, 1.85%), pentanucleotide (10,975 motifs, 0.57%), and hexanucleotide (9,930 motifs, 0.51%) repeats. With an increase in the repeat motif length, the number of SSRs decreased. Among the mononucleotides (Table 4), A/T repeats were the predominant type (1,174,392 motifs, 97.84%). Among the dinucleotides (Table 5), AG/CT (277,157 motifs, 49.74%) and AT/AT repeats (228,679 motifs, 41.04%) were dominant, followed by AC/GT repeats (49,972 motifs, 8.97%), whereas CG/GC repeats (1410 motifs, 0.25%) were the lowest. Among the trinucleotides (Table 6), the most frequent motif was AAT/ATT repeats (47,924 motifs, 37.95%), followed by AAG/CTT (26,511 motifs, 20.99%) and ACC/GGT (22,235 motifs, 17.61%) repeats. ACG/CGT repeats (725 motifs; 0.57%) were the least frequent trinucleotide motifs. The longest tetra-, penta-, and hexanucleotide SSR repeats were AAAT/ATTT (23,406 motifs, 65.22%), AAAAT/ATTTT (2,951 motifs, 26.89%), and AAAAAT/ATTTTT (1187 motifs, 11.95%), respectively (Tables 7–9). To provide more information for SSR primer verification in future research, 49,046 SSRs (tr- and tetranucleotide) were suited to the designed primers. Primer information is presented in Supplementary Table 1.

## 4. Discussion

In the genus *Camellia*, the genomes of *C*. *sinensis* and *C*. *oleifera* have been sequenced and assembled [53, 54]. The genome size of *C*. *sinensis* ranged from 3,062.62 Mb (*C*. s*inensis* var. assamica) to 3,113.46 Mb (*C*. *sinensis* isolate G240). The CNC genome size was close to that of *C*. *oleifera*, which was 2889.51 Mb [54]. However, it was smaller than that of *C*. *sinensis*. The GC content of *C*. *oleifera* was 34.5189% [54]. The median GC content of *C*. *sinensis* was 38.5319% in the NCBI genome database. The GC content of CNC was close to that of *C*. *oleifera* but lower than that of *C*. *sinensis*. The result showed that *C*. *oleifera* is closer to CNC than *C*. *sinensis* in phylogenetic relationships, which is consistent with previous studies [55]. The genome assembly strategies of other species in the genus *Camellia* can be applied to CNC, such as Illumina combined with PacBio (or Oxford Nanopore Technologies) and Hi-C-based assembly, and genome assembly should be as difficult as *C*. *oleifera*, but less difficult than *C*. *sinensis*. The genome size estimated using NGS becomes more difficult in cases of high heterozygosity and high duplication, which can be further verified by constant-value (C-value) using flow cytometry. The motifs of SSRs including A or T were more abundant than those including C or G, the characteristics and distributions of which were similar to those reported in previous studies on *C*. *sinensis* [41]. Further validation studies of SSR markers are needed for the CNC population.

In the current study, the whole genome of CNC was sequenced using NGS for the first time, which will play an important role in future whole-genome sequencing projects. Statistical analysis of the differences in the quantity and motifs of SSRs provided a foundation for the further construction of high-density genetic maps of CNC. The wild CNC is an endangered plant in China. Therefore, the CNC genome survey will have important ecological significance.

## 5. Conclusions

In the present study, an approximate genome size of 2,778.82 Mb of CNS was estimated using the 21-mer analysis, with heterozygosity and repetition rates of 1.42% and 65.27%, respectively. The results showed the genomic characteristics of CNS were similar to those of *C*. *oleifera*. In total, 1,940,616 SSRs were identified in the genome data. We believe these results will provide meaningful data for conducting further genomic studies and a useful basis for the development of novel molecular markers. Hence, novel state-of-the-art genetic techniques, such as Illumina combined with PacBio HiFi and Hi-C-based assembly, need to be developed to obtain chromosomal-level scaffolding genomes.

## Figures and Tables

**Table 1:** Reads statistics of CNC.

Raw reads base (bp)	Raw reads num	Clean reads base (bp)	Clean reads num	Q20 (%)	Q30 (%)	GC (%)
382, 206, 016, 200	2, 548, 040, 108	343, 061, 954, 700	2, 287, 079, 698	95.67	89.52	37.00

Q20, percentage of bases with quality value ≥20; Q30, percentage of bases with quality value ≥30; GC, GC content.

**Table 2:** CNC genome estimation based on K-mers analysis.

K-mers	K-mers depth	Genome size (bp)	Error rate (%)	Duplication rate (%)	Heterozygous rate (%)	Repeat rate (%)
19	103	2, 778, 996, 247	0.223	0.849	1.46	70.41
21	101	2, 778, 823, 868	0.248	0.706	1.42	65.27
23	99	2, 775, 979, 652	0.258	0.729	1.39	61.40

**Table 3:** Statistics of the assembled CNC genome.

	Total length (bp)	Total number	Max length (bp)	N50 length (bp)	GC content (%)
Contig	2, 844, 296, 380	9, 994, 482	73, 907	649	36.00
Scaffold	2, 910, 885, 755	9, 399, 197	88, 303	869	34.00

**Table 4:** Statistics of mononucleotide 1, 200, 317 motifs.

Repeat motif	Number of repeats							Total	Ratio (%)
	10	11	12	13	14	15	>15		
A/T	351876	203253	134009	95773	75424	63479	250578	1174392	97.84
C/G	5260	4626	3724	2882	1948	1269	6216	25925	2.16

**Table 5:** Statistics of dinucleotide 557, 218 motifs.

Repeat motif	Number of repeats							Total	Ratio (%)
	6	7	8	9	10	11	>11		
AG/CT	64546	42040	30640	22676	17001	13126	87128	277157	49.74
AT/AT	44869	29446	26371	28751	31286	28220	39736	228679	41.04
AC/GT	15516	8511	5931	4365	3118	2376	10155	49972	8.97
CG/CG	836	340	146	53	23	11	1	1410	0.25

**Table 6:** Statistics of trinucleotide 126, 286 motifs.

Repeat motif	Number of repeats							Total	Ratio (%)
	5	6	7	8	9	10	>10		
AAT/ATT	18737	9697	5013	2873	2015	1787	7802	47924	37.95
AAG/CTT	11174	5169	2875	1947	1439	1139	2768	26511	20.99
ACC/GGT	10235	5006	2968	1838	994	567	627	22235	17.61
AAC/GTT	4654	2212	1214	676	418	285	424	9883	7.83
ATC/ATG	4353	1767	903	546	341	267	436	8613	6.82
AGG/CCT	2731	1177	645	484	318	241	246	5842	4.63
AGC/CTG	1194	402	214	90	35	11	16	1962	1.55
CCG/CGG	779	323	136	81	31	12	10	1372	1.09
ACT/AGT	608	252	117	74	35	36	97	1219	0.97
ACG/CGT	409	147	93	36	19	12	9	725	0.57

**Table 7:** Statistics of tetranucleotide 35, 890 motifs.

Repeat motif	Number of repeats							Total	Ratio (%)
	5	6	7	8	9	10	>10		
AAAT/ATTT	17047	4468	1220	389	160	65	57	23406	65.22
AAAC/GTTT	1673	733	307	122	40	22	3	2900	8.08
AAAG/CTTT	1254	487	202	134	49	20	10	2156	6.01
AGAT/ATCT	756	420	280	162	66	47	36	1767	4.92
ACCC/GGGT	973	315	106	30	2	1	0	1427	3.98
ACAT/ATGT	418	195	124	59	40	12	21	869	2.42
AATT/AATT	534	143	41	14	4	3	0	739	2.06
AGGG/CCCT	288	160	88	37	17	10	5	605	1.69
ACAG/CTGT	298	106	45	12	1	0	1	463	1.29
ACTC/AGTG	199	99	48	12	5	2	0	365	1.02
Others	719	174	60	19	11	3	2	988	2.75

**Table 8:** Statistics of pentanucleotide 10, 975 motifs.

Repeat motif	Number of repeats							Total	Ratio (%)
	5	6	7	8	9	10	>10		
AAAAT/ATTTT	2321	458	123	32	13	3	1	2951	26.89
AAAAG/CTTTT	1143	355	99	17	3		0	1617	14.73
AAAAC/GTTTT	948	327	93	34	12	3	0	1417	12.91
AAACC/GGTTT	729	293	84	29	6	3	0	1144	10.42
AACCC/GGGTT	212	125	49	9	1		1	397	3.62
AGCCG/CGGCT	184	75	21	4	2	1	0	287	2.62
AAGAG/CTCTT	144	48	10	5	1	1	0	209	1.90
AATAT/ATATT	110	33	10	10	4	1	0	168	1.53
AAATT/AATTT	122	20	3	0	0	1	0	146	1.33
Others	1880	538	151	45	14	9	2	2639	24.05

**Table 9:** Statistics of hexanucleotide 9, 930 motifs.

Repeat motif	Number of repeats							Total	Ratio (%)
	5	6	7	8	9	10	>10		
AAAAAT/ATTTTT	795	216	128	41	4	1	2	1187	11.95
AAAAAC/GTTTTT	509	186	89	27	0	1	4	816	8.22
AAAAAG/CTTTTT	441	141	59	16	1	0	0	658	6.63
AGAGGG/CCCTCT	215	67	19	1	0	0	1	303	3.05
ACCGCC/CGGTGG	147	64	26	16	0	0	0	253	2.55
ACCTCC/AGGTGG	135	54	23	2	1	0	0	215	2.17
AACCCT/AGGGTT	114	52	16	7	1	0	0	190	1.91
AAAATC/ATTTTG	106	31	20	8		0	0	165	1.66
ACCATC/ATGGTG	103	34	12	3	2	1	1	156	1.57
AAAGAG/CTCTTT	103	35	13	1	0	0	0	152	1.53
Others	3777	1342	464	214	27	3	8	5835	58.76

## Data Availability

The following information was supplied regarding the deposition of DNA sequences: the raw data can be obtained from the Sequence Read Archive at NCBI under accession numbers SRR19315149. The associated BioProject, Bio-Sample numbers are PRJNA839723, SAMN28548419, respectively.

## Supplementary Materials

Supplementary Materials Supplementary Table 1. SSR primers pairs of the CNC genome.
